# Anastrozole-induced pulmonary cryptococcosis in a patient with early breast cancer: A case report

**DOI:** 10.1097/MD.0000000000018688

**Published:** 2020-01-10

**Authors:** Min Wei, Yu-Rong Xu, Kui Liu, Peng Wen

**Affiliations:** a Department of Respiratory Medicine, Shandong Chest Hospital Affiliated to Shandong University, Jinan,; b Department of Orthopedics, Shijiazhuang Third Hospital, Shijiazhuang,; c Department of Respiratory Medicine, Qilu Hospital of Shandong University, Jinan, China.

**Keywords:** anastrozole, estrogen, immunodeficiency, pulmonary cryptococcosis

## Abstract

**Introduction:**

Estrogen is a key factor in breast cancer carcinogenesis, and reductions in its synthesis can decrease breast cancer risk. Anastrozole can reduce plasma estrogen levels by inhibiting the enzyme aromatase, and is approved for adjuvant treatment of breast cancer. We report a case of pulmonary cryptococcosis in a patient who was treated with anastrozole for an early-stage tumor. This case is of special interest because the patient achieved a better curative effect after the administration of anastrozole was discontinued.

**Patient concerns:**

A 61-year-old woman was found to have multiple pulmonary nodules on chest computed tomography (CT) after being treated for 5 months with anastrozole as an adjuvant breast cancer therapy. A biopsy of the largest lesion of the right lung showed cryptococcus fungal bodies with granulomatous inflammation, so the patient was diagnosed with pulmonary cryptococcosis. She was treated with fluconazole (400 mg/day) for 1 month, but a follow-up CT scan of chest showed no improvement.

**Diagnosis:**

Pulmonary cryptococcosis.

**Interventions:**

Because the pulmonary cryptococcosis was not improving, the administration of anastrozole was discontinued. Fluconazole was continued.

**Outcomes:**

The pulmonary lesions diminished in size 2 months after discontinuing anastrozole. The patient continued taking fluconazole for a total of 6 months without re-administration of anastrozole, and the lesions of pulmonary cryptococcosis almost disappeared.

**Conclusion:**

This case of pulmonary cryptococcosis may have been induced by a decrease in estrogen level caused by the aromatase inhibitor, anastrozole. Treatment of pulmonary cryptococcosis with concurrent anastrozole use may be ineffective, and it may be better to discontinue the aromatase inhibitor.

## 1 Introduction

Estrogen is a key factor in breast cancer carcinogenesis, and reducing estrogen synthesis can decrease breast cancer risk. Estrogen production is driven by the enzyme aromatase, which is responsible for peripheral conversion of androgens to estrogens. Anastrozole is a non-selective aromatase inhibitor approved for adjuvant treatment of early-stage, hormone receptor-positive breast cancer in postmenopausal women.^[1]^ Anastrozole reduces plasma estrogen levels by inhibiting aromatase. It requires long-term use, and its most important adverse effects are an increased risk of bone fractures and myalgia/arthralgia.^[2]^ Other adverse events have been less frequently reported.

Pulmonary cryptococcosis is known to occur particularly frequently in immunocompromised hosts.^[3]^ It is prevalent in patients with a malfunction in the immunity mediated by cells, such as in acquired immunodeficiency syndrome, transplant-related immunosuppression, corticosteroid therapy, chemotherapy, neoplasms, and lymphoproliferative disorders.^[3,4]^ However, cryptococcosis can also occur in patients who have not been found to have immunodeficiency. It has further been reported that estrogen plays an important role in the regulation of the immune system by inducing direct effects on multiple cell types.^[5]^ Emerging data from the literature suggest that estrogen deficiency is associated with increased infection.^[6,7]^

Herein, we report a case of anastrozole-related infection, suggesting a possible role of the immune system in anastrozole-related side effects. We also review the case in the context of related published literature. Written informed consent was obtained from the patient for the publication of this case study.

## 2 Case report

A 60-year-old woman underwent left mastectomy and regional lymph node dissection for a 1.2 × 0.7 mm mass in July 2017. The invasive ductal carcinoma was estrogen receptor (ER) positive (90%), progesterone receptor (PR) positive (60%), and human epidermal growth factor receptor 2 (HER2) (1+); no metastatic lymph nodes were found. She was an otherwise healthy postmenopausal woman. The tumor was in the early stage, so radiotherapy and chemotherapy were not performed. The patient did not have any known immunodeficiency. Because she was postmenopausal and ER+, following surgery, 1 mg/day of anastrozole was started in August 2017.

She was admitted to our hospital for multiple pulmonary nodules on chest computed tomography (CT) (Fig. 1A and B) in April 2018. The lesions were found mainly in the subpleural regions, and the largest lesion was 14 mm in diameter. The patient had no respiratory symptoms, vital signs were stable, and physical examination revealed a good nutritional state, with normal respiration and no lymph node enlargement. No positive signs were detected after admission. Initial investigations such as total leukocyte count, white blood cell differential count, and renal and liver function tests were within normal limits, and C-reactive protein and erythrocyte sedimentation rate were both within normal limits. She tested negative for anti-HIV antibodies. Flow cytometry assays to assess her T and B lymphocyte levels produced normal results.

**Figure 1:**
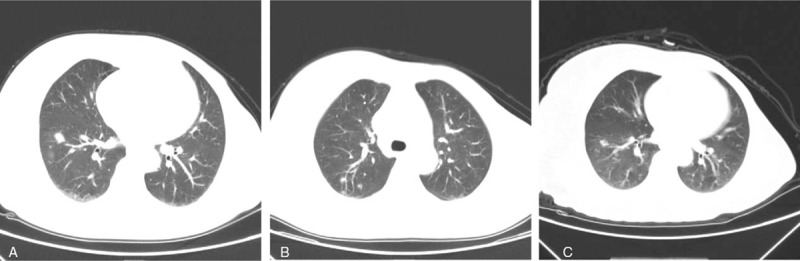
Findings of a chest CT. (A,B): The largest lesion was 14 mm in diameter. C: Findings of a chest CT after discontinuing the administration of anastrozole for 2 months.

**Figure 2:**
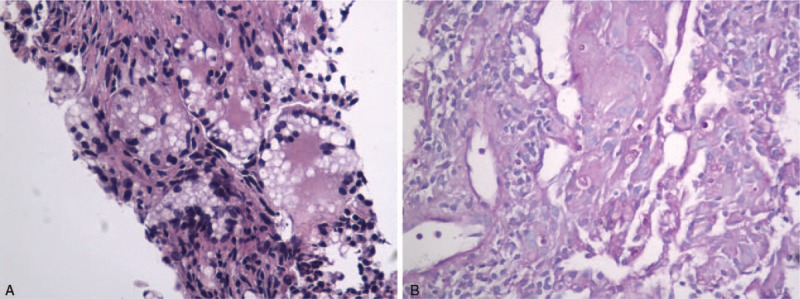
Pathological findings of biopsy specimens from the largest lesion of the patient's right lung. Granulomatous change, fungus bodies of cryptococcus. A: HE staining, ×400 B: PAS staining, ×400.

The patient was treated with antibiotics (amoxicillin and levofloxacin), but there was no clinical improvement. She was suspected to have lung metastasis from breast cancer. Serum cryptococcal antigen, measured by enzyme-linked immunosorbent assay (ELISA), was positive (46.05 μg/L). A biopsy of the largest lesion of the right lung showed cryptococcus fungal bodies with granulomatous inflammation (Fig. 2A and B). Lumbar puncture was performed, but no pathogens were found in cerebrospinal fluid (CSF) by Gram stain, and CSF cryptococcal antigen test was negative. Therefore, the patient was diagnosed with pulmonary cryptococcosis. She was treated with fluconazole (400 mg/day) for 1 month, but a follow-up CT scan of chest showed no improvement. Serum cryptococcal antigen was positive (44.13 μg/L). Because the cryptococcal antigen titer had not improved significantly compared with 1 month previously, the administration of anastrozole was discontinued. Two months later, the pulmonary lesions had diminished in size (Fig. 1C). The patient continued fluconazole for a total of 6 months without re-administration of anastrozole, and the lesions of pulmonary cryptococcosis almost disappeared. The antigen titration gradually declined and was finally found to be negative. Subsequently, this patient switched to tamoxifen to continue treatment for breast cancer because of the importance of estrogen reduction. She was also told to avoid possible sources of cryptococcus, such as pigeon droppings. No recurrence of cryptococcosis has been found.

## 3 Discussion

In this case report, we summarized two important clinical observations:

(1) one patient who received treatment with aromatase inhibitor developed pulmonary cryptococcosis; and
(2) fluconazole treatment was ineffective when combined with anastrozole.

Infections (including viral, bacterial, and fungal) associated with anastrozole have been reported in randomized controlled trials^[8–10]^; however, the incidence varies among clinical trials, and the trials have not had the power to reveal an increased risk of infections. Additionally, no systematic synthesis of the data to determine the association of infections with anastrozole has been attempted.

Fluconazole is an inhibitor of the cytochrome P450 family of enzymes. Drug interactions with fluconazole may be related to its inhibition of hepatic drug metabolism, specifically when administered with phenytoin, terfenadine, warfarin, cyclosporin A, and tolbutamide.^[11–16]^ Anastrozole is mainly metabolized in the liver by hydroxylation, glucuronidation, and dealkylation. A cytochrome mediated drug–drug interaction might cause plasma anastrozole concentrations to rise, further lowering the patient's estrogen levels. Anastrozole has also been shown to inhibit the reactions catalyzed by cytochromes P450, 1A2, 2C8/9, and 3A4.^[17]^ Unlike anastrozole, fluconazole is metabolically stable, with renal excretion accounting for 80% of the elimination as unchanged drug.^[18]^ Moreover, no pharmacological studies have shown that co-administration of anastrozole and fluconazole resulted in a significant decrease in the activity of fluconazole. In our case, the clinical relevance of the drug–drug interaction between anastrozole and fluconazole remains uncertain. In addition, the literature shows that fluconazole does not affect estrogen levels.^[11]^

Cryptococcosis has been reported in immunocompetent patients. The mechanism of pulmonary cryptococcosis in patients with normal cellular immunity is not well established. The regulation of the immune response to infection is a complex interplay of multiple factors. In so-called “not immunosuppressed” patients, it is possible that—in some cases—an unclear immunodeficiency is clinically not apparent and/or not detected because of technical limitations. Previous research has shown that autoantibodies against interferon (IFN)-γ were detected in 88% of Asian adults who had normal cellular immunity with multiple opportunistic infections,^[19]^ but the level of anti-IFN-γ antibody was negative in this case. A strong relationship between estrogen and the immune system has been reported in the literature. Moreover, in addition to its essential role in sexual development and reproduction in females, estrogen is involved in a wide range of physiological processes in different tissues.^[20]^ Previous reports have shown that estrogen influences the functioning of the immune system. Estrogen has been shown to markedly attenuate the release of pro-inflammatory mediators such as tumor necrosis factor alpha, interleukin (IL)-1 beta, and IL-6 from both neutrophils and macrophages in rats, mice, and humans.^[21,22]^ In particular, estrogen has clearly been shown to interact with nuclear factor kappa B signaling to limit inflammatory activity; there is also accumulating evidence for a role of the hormone in regulating the anti-inflammatory/pro-resolution protein annexin A1.^[22]^

Emerging data from the literature suggest that the immune system demonstrates remarkable sex differences. The outcome and survival rates from infections or sepsis are sometimes better in females than in males.^[23]^ In addition, it is reported that estrogen inhibits growth of cryptococcus neoformans in vitro.^[4]^ This case is an otherwise healthy postmenopausal woman. She did not present any known immunodeficiency. Based on previous findings that chronic low levels of estrogen in the body may be associated with cryptococcal infections, estrogen may play a key role in this case. Some studies have shown that the level of cryptococcal antigen titer is often significantly correlated with the severity of disease: progressive disease is usually accompanied by increasing antigen titers, while declining titers are usually associated with clinical improvement. The cryptococcal antigen latex agglutination test appears to have both diagnostic and prognostic value.^[24,25]^ In our case, the declining cryptococcal antigen titer and the improvement achieved after drug withdrawal suggest a probable correlation between anastrozole and pulmonary cryptococcosis.

Because of the importance of lowering estrogen levels in the treatment of ER-positive breast cancer patients, switching to drugs such as tamoxifen or toremifene that are not aromatase inhibitors may be a better option in situations like this. In the present case, pulmonary cryptococcosis occurred in a patient treated with anastrozole. To the best of our knowledge, this is the first report of a pulmonary cryptococcosis infection occurring in a patient treated with an aromatase inhibitor, which can effectively reduce the estrogen level in the body that causes breast cancer cell growth.

## 4 Conclusion

Because anastrozole should be taken for at least 5 years, dealing with the side effects has become a major issue. Anastrozole -related pulmonary cryptococcosis is a rare condition, but as in this case, discontinuing aromatase inhibitor may be considered as a treatment option. Further studies and case reports about this adverse effect can provide more information in the future.

## Author contributions

**Conceptualization:** Peng Wen.

**Data curation:** Min Wei, Yu-Rong Xu, Kui Liu.

**Formal analysis:** Min Wei, Peng Wen.

**Investigation:** Min Wei, Peng Wen, Kui Liu.

**Project administration:** Yu-Rong Xu, Kui Liu.

**Software:** Min Wei, Yu-Rong Xu, Kui Liu.

**Supervision:** Peng Wen.

**Writing – original draft:** Min Wei, Kui Liu.

**Writing – review & editing:** Peng Wen.

Peng Wen orcid: 0000-0001-8213-8641.
